# Genome sequence of the marine bacterium *Roseobacter* sp. EG26, isolated from the octocoral *Eunicella gazella*, suggests aptitude for a host-associated lifestyle

**DOI:** 10.1128/mra.00430-25

**Published:** 2025-07-14

**Authors:** Tina Keller-Costa, Selene Madureira, Ana S. Fernandes, Lydia Kozma, Jorge Gonçalves, Cristina Barroso, Ali Budhi Kusuma, Conceição Egas, Rodrigo Costa

**Affiliations:** 1 Institute for Bioengineering and Biosciences (IBB) and Institute for Health and Bioeconomy (i4HB), Instituto Superior Técnico, Universidade de Lisboa 37809, Lisbon, Portugal; 2 Department of Bioengineering, Instituto Superior Técnico, Universidade de Lisboa, Lisboa, Portugal; 3 École Polytechnique Fédérale de Lausanne 27218, Écublens, Switzerland; 4 Algarve Centre of Marine Sciences (CCMAR), Campus de Gambelas, Faro, Portugal; 5 Biocant—Transfer Technology Association, BiocantPark, Cantanhede, Portugal; 6 Center for Neuroscience and Cell Biology (CNC), Rua Larga - Faculdade de Medicina, University of Coimbra 37829, Coimbra, Portugal; California State University San Marcos, San Marcos, California, USA

**Keywords:** *Roseobacteraceae*, *Alphaproteobacteria*, coral, holobiont, symbiosis

## Abstract

We present the genome sequence of the octocoral-associated *Roseobacter* sp. EG26. We highlight features related to type II, III, IV, and VI secretion systems, ankyrin-repeat proteins, and taurine degradation, suggesting a preference for a host-associated lifestyle. Strain EG26 also possesses genes for the degradation of phenolic compounds with bioremediation potential.

## ANNOUNCEMENT

*Alphaproteobacteria* of the *Roseobacteraceae* family are commonly found in association with marine eukaryotes across ocean basins and may constitute up to 50% of the coral mucus microbiome (1). Some *Roseobacteraceae* bacteria seem to play a role in coral reproduction due to their intimate association with coral larvae and high abundance after coral spawning (2, 3). Although *Roseobacteraceae* spp. are consistent members of healthy corals, they have also been shown to increase in abundance during dysbiosis (4), and current understanding of their function in the coral holobiont is limited.

We report the genome of *Roseobacter* sp. EG26 isolated from a healthy-looking *Eunicella gazella* specimen (Cnidaria, Anthozoa, Octocorallia), collected by SCUBA diving at 18 m depth in the Northeast Atlantic off the coast of Faro, Portugal (Pedra da Greta: latitude 36.979778 N, longitude 7.98911 W) on 21 April 2021. The specimen was processed immediately after sampling. A microbial cell suspension was retrieved from 1 g of coral soft tissue by mortar-and-pestle homogenization in 9 mL sterile Ca^2+^ and Mg^2+^-free artificial seawater as described earlier (5, 6). Serial dilutions were prepared, plated on Marine Agar (MA), and incubated at 24°C for a week (6). Single colonies were streaked for purity on MA plates. Genomic DNA was extracted from a cell pellet obtained by centrifugation (10,000 rcf, 30 min) of a pure culture freshly grown in Marine Broth, using the Wizard Genomic DNA Purification kit (Promega, USA). Genome sequencing was performed at GENOINSEQ (Biocant, Cantanhede, Portugal). A genome library was prepared with the Nextera XT DNA Library Preparation Kit (Illumina), and the genome was paired-end sequenced (average read length, 269/272 bp) on an Illumina MiSeq apparatus with the MiSeq Reagent Kit v3 (600 cycles). Default parameters were used for all software. Raw reads were imported to KBase (7), merged, and quality-checked using FastQC v0.12.1. Low-quality reads were removed using Trimmomatic v.0.36 (8), and trimmed sequence reads were assembled using SPADES v3.15.3 (9). Contigs below 1,000 bp were removed. Genome completeness and contamination were estimated with CheckM v1.0.18 (10) and whole genome-based taxonomy assigned with GTDB-Tk v2.3.2 (11). Contigs were annotated using the NCBI Prokaryotic Genome Annotation Pipeline (PGAP v.6.10) (12), and protein family (Pfam) domains were further annotated using our in-house Melange pipeline (https://github.com/sandragodinhosilva/melange).

Table 1 presents basic genome features of *Roseobacter* sp. EG26. Phylogenomic analysis identified *Roseobacter insulae* YSTF-M11 (GCF_019375555.1)—isolated from tidal flats in the Yellow Sea (13)—as its closest relative. However, the genomes of strains EG26 and YSTF-M11 shared only 77.07% average nucleotide identity as calculated using FastANI v0.1.3 (14), suggesting that strain EG26 may represent a novel species. The EG26 genome harbors multiple Pfam features related to type II, III, IV, and VI secretion systems, serine/threonine protein kinases, ankyrin-repeat proteins, and taurine degradation, indicating affinity for a host-associated lifestyle (4, 15, 16). Moreover, the strain holds the genomic blueprint for the biosynthesis of vitamins B1, B6, B7, B9, and B12. Genes encoding enzymes involved in the biodegradation of phenolic compounds, such as methane/phenol/toluene hydroxylases (PF02332.22), were found, suggesting a potentially beneficial (bioremediation) role for this strain—e.g., under ocean acidification scenarios, where the accumulation of phenolic compounds is expected to increase (17).

**TABLE 1:** General features of the *Roseobacter* sp. EG26 genome reported in this study

							Estimate (%) of		Number of		Count by									
**Genome size (bp**)	**GC-content (%**)	**Genome coverage (x**)	**Number of contigs**	**Contig N50 (bp**)	**Number of reads**	**Average read length (bp**)	**Completeness (%**)	**Contamination (%**)	**Coding sequences^*a*^**	**Genes^*a*^**	**RNA^*a*^**	**rRNA^*a*^**	**tRNA^*a*^**	**ncRNA^*a*^**	**COG^*b*^**	**Pfam^*b*^**	**GenBank accession number**	**SRA accession number**	**Bioproject accession number**	**Biosample accession number**
5,094,239	57.1	202.7	46	412,916	2 × 1,805,825	269/272	99.57	0.06	4,906	4,957	51	3	44	4	3,707	8,481	JBNBLH000000000	SRR32940153	PRJNA1242562	SAMN47596050

^*a*^ Annotation was performed by the NCBI Prokaryotic Genome Annotation Pipeline (PGAP v. 6.10): https://www.ncbi.nlm.nih.gov/genome/annotation_prok/.

^*b*^ The Melange pipeline (https://github.com/sandragodinhosilva/melange) was used to perform Protein family (Pfam) and Clusters of Orthologous Groups of proteins (COG)-based annotations. The corresponding data are available on Zenodo under DOI: https://doi.org/10.5281/zenodo.15196899.

## Data Availability

The genome sequence of Roseobacter sp. strain EG26 has been deposited at DDBJ/ENA/GenBank under the BioProject PRJNA1242562, SRA accession number SRR32940153, and the Whole Genome Shotgun accession number JBNBLH000000000. The version described in this paper is version JBNBLH010000000. The Melange results of the genome’s Pfam and COG annotations are available on Zenodo under DOI: https://doi.org/10.5281/zenodo.15196899. Further details can be found in Table 1.
